# Trends in prevalence of obesity and its association with hypertension across socioeconomic gradients in rural Yunnan Province, China

**DOI:** 10.1186/s12872-024-03741-1

**Published:** 2024-01-28

**Authors:** Xia Wu, Guohui Li, Lan Liu, Yi Zhao, Allison Rabkin Golden, Le Cai

**Affiliations:** 1 Yunnan Provincial Key Laboratory of Public Health and Biosafety & School of Public Health, Kunming Medical University, 1168 Yu Hua Street Chun Rong Road, Cheng Gong New City, Kunming, 650500 China; 2 The Second Affiliated Hospital of Kunming Medical University, 374 Yunnan- Myanmar Avenue, Wu Hua District, Kunming, 650106 China; 3 The First Affiliated Hospital of Kunming Medical University, 295 Xi Chang Raod, Wu Hua District, Kunming, 650031 China

**Keywords:** Obesity, Hypertension, Temporal trend, Socioeconomic status, China

## Abstract

**Background:**

This study aimed to uncover the changing prevalence of obesity and its association with hypertension across socioeconomic gradients in rural southwest China.

**Methods:**

Data were collected from two cross-sectional health interviews and surveys from 2011 to 2021 among individuals aged ≥ 35 years in rural China. Each participant’s height, weight, waist circumference, and blood pressure were measured. The overall prevalence of obesity, central obesity, and hypertension was directly standardized by age based on the total population of the two surveys. Multivariate logistic regression was used to analyze the association between obesity and prevalence of hypertension and an individual socioeconomic position (SEP) index was constructed using principal component analysis.

**Results:**

From 2011 to 2021, the prevalence of obesity, central obesity, and hypertension increased substantially, from 5.9%, 50.2%, and 26.1–12.1%, 58.0%, and 40.4% (*P* < 0.01), respectively. These increasing rates existed in all subcategories, including sex, age, ethnicity, education, annual household income, access to medical services, and SEP (*P* < 0.05). In both 2011 and 2021, lower education level and poor access to medical services correlated with higher prevalence of central obesity, while higher SEP correlated with higher prevalence of obesity and central obesity (*P* < 0.01). Prevalence of obesity was higher in the Han ethnicity participants and individuals with poor access to medical services than in their counterparts (*P* < 0.01). Whereas the prevalence of central obesity was lower in Han participants than in ethnic minority participants in 2011 (*P* < 0.01), this trend reversed in 2021 (*P* < 0.01). A positive relationship between annual household income and prevalence of obesity and central obesity was only found in 2021 (*P* < 0.01). Obese and centrally obese participants were more likely to be hypertensive in both survey years (*P* < 0.01).

**Conclusions:**

Future interventions to prevent and manage obesity in rural China should give increased attention to high income, less educated, poor access to medical services, and high SEP individuals. The implementation of these obesity interventions would also help reduce the prevalence of hypertension.

## Background

Obesity is a chronic disease whose origination and development is influenced by both genetic and environmental factors [1]. It has became a pressing global public health concern [2], and is also a main contributing factor to chronic non-communicable diseases (NCDs), such as cardiovascular diseases (CVD), hypertension, and certain types of cancer, among others [3]. Over the last 40 years, there has been a threefold increase in obesity rates worldwide [4], and no country is experiencing a decline in obesity prevalence [5]. From 1975 to 2019, the prevalence of obesity in adult men and women rose from 3.2% and 6.4–12.3% and 16.2%, respectively [6, 7]. Meanwhile, increasing physical inactivity and unhealthy dietary patterns are causing a simultaneous rise in central obesity prevalence worldwide — between 1985 and 1999 and 2010–2014, the worldwide prevalence of central obesity increased from 31.3–48.3% [3] — and it has caused significant financial strain on public health systems worldwide.

There has been a significant increase in both obesity and central obesity rates within China [8], with obesity increasing from 2.1 to 16.3% in men and 2.5–12.4% in women between 1975 and 2014 [6], and central obesity increasing from 19.8 to 43.2% between 1993 and 2011 [9]. Adults living in rural areas experienced a faster escalation of both obesity and central obesity prevalence in contrast to those living in cities [9–11].

Correspondingly, it has an increase in the occurrence of chronic diseases related to obesity in China [12], including hypertension, which is not only is a main risk factor for NCDs, but a significant medical issue itself [13]. Specifically, from 1959 to 2016, hypertension prevalence in people aged ≥ 18 years increased from 5.1–25.2% [14]. Obesity has been suggested to account for 75% of hypertension cases in population-based studies [15].

Studies have shown that measures of socioeconomic status (SES) are linked to both obesity and central obesity [16–19]. Namely, numerous studies have shown that low SES is related to a higher possibility of being obese and centrally obese in high-income countries. By comparison, low- and middle-income countries have shown mixed associations, both positive and negative, between SES and obesity [18]; according to research, as low- and middle-income countries experience an increase in gross domestic product (GDP), the relationship SES and obesity shifts from being positively correlated to negatively correlated [16]. In China, income is positively correlated with obesity prevalence, whereas the association of education with obesity is diverse, with men showing a positive association and women exhibiting an inverse association [8, 17, 19].

However, research on socioeconomic determinants of obesity is currently lacking in rural regions of China. Further, little study has been carried out to evaluate the impact of individual SES on changes in obesity prevalence over time. Thus, this study aimed to investigate the correlation between socioeconomic factors and changes in obesity prevalence from 2011 to 2021, as well as to explore the relation between obesity and hypertension within adults in rural areas of southwest China.

## Methods

### Study area and population

Data for this study were obtained from two health surveys conducted within communities through cross-sectional methods, which involved interviews and physical examinations. The surveys were performed in three rural regions of Yunnan Province, spanning two distinct time periods: 2010–2011 and 2020–2021. Situated in the southwest region of China, Yunnan Province is among the country’s provinces with the lowest income levels. It has a population of 46.9 million, and is home to the most ethnic diversity of any province in China, where 25 different ethnic groups reside. Ethnic minorities account for 38.2% of Yunnan’s total population and have a wide range of SES. The previous studies indicated that ethnicity significantly impact prevalence of obesity and hypertension in rural Yunnan province [20]. Thus, ethnicity may be an important SES consideration in the field of obesity and hypertension study in Yunnan province.

In both 2011 and 2021, 129 counties were categorized into low, medium, and high wealth groups based on their per capita GDP. Subsequently, a total of three counties were randomly chosen from each of these groups during each survey year. The study participants aged ≥ 35 years from the chosen three rural counties were selected using a uniform three-stage stratified random sampling method in both of these two cycles. Further detail about the sampling method employed has been outlined in previous studies [21].

### Data collection and measurement

In both the 2011 and 2021 surveys, each participant was interviewed face to face by trained investigators using a standardized and pretested questionnaire. The questionnaire included information about demographic features (sex, age, ethnicity, education, and annual household income), diagnosis, treatment, and self-reported family history of hypertension. All subjects had their blood pressure (BP) and anthropometric measurements recorded.

According to American Heart Association (AHA) [22] recommendations, a total of three blood pressure readings were recorded using a mercury sphygmomanometer. Further detail about the measurement method employed has been described in previous studies [21, 23].

The study followed a standardized protocol to determine participants’ height, weight, and waist circumference (WC). Further detail about the measurement method employed also has been described in previous studies [21, 23].

### Definitions

The body mass index (BMI) is a calculation obtained by dividing a person’s weight in kilograms by the square of their height in meters (kg/m^2^).WC and BMI are two standards used to classify general obesity and central obesity. Obesity was defined as BMI ≥ 28 kg/m^2^, and central obesity was defined as WC ≥ 80 cm in women and WC ≥ 90 cm in men. These definitions of obesity and central obesity were based on the World Health Organization (WHO) [24] and the International Diabetes Federation (IDF) for those of Asian background, respectively [25].

Hypertension was defined as a mean systolic blood pressure (SBP) ≥ 140 mm Hg and/or a mean diastolic blood pressure (DBP) ≥ 90 mm Hg, currently being on antihypertensive therapy, and/or having previously received a hypertension diagnosis from a licensed physician.

Based on the 56 ethnic groups in China, they can be divided into two major categories: Han majority and ethnic minority. Ethnic minority was used to refer to individuals who had a religion, culture, and/or language that differed from those of the Han population, which constituted the majority. Illiteracy is characterized by the absence of the ability to comprehend written language or to produce basic written communication related to everyday activities. The education level of the participants was divided into two categories: those who were illiterate and those who had completed primary education (grades 1–6) or above. The participants were separated into two categories based on their annual household income: low and high, using the median value as the threshold. Access to medical services was defined as good when walking time to the nearest village hospital was ≤ 30 min and poor when walking time to the nearest village hospital > 30 min.

### Statistical analysis

We analyzed the data using SPSS 22.0 software after entering it twice into an EpiData 3.1. Categorical variables were reported as counts and percentages. The total population of the two surveys was used as standardization population, and the overall prevalence of obesity, central obesity, and hypertension was calculated within each age range stratified by ethnicity, income, education, access to medical services, and SEP, and then applied that to the population we standardized too. Categorical variables were compared across survey years using chi-square tests, while ordered variables were compared using chi-square tests for trend. Logistic regression was used to analyze the relationship between the prevalence of obesity and central obesity with hypertension. Their associations were expressed as odds ratios and 95% confidence intervals (95% CI). The p-value for the two-sided test was less than 0.05, indicating a statistically significant difference.

A composite index was used as a proxy for individual socioeconomic position (SEP), and principal components analysis (PCA) was used to construct the SEP index. The SEP for this estimation included three socioeconomic indicators—education, annual household income, and access to medical services. The indicators used the following conditions: (1) Correlation refers to the correlation coefficient among indicators and is ≥ 0.3; (2) Reasonable structure refers to the value of the Kaiser-Meyer-Olkin (KMO) and is ≥ 0.70 when analysis is on the overall structure and ≥ 0.5 when analysis is on a single indicator; (3) The Bartlett’s test of sphericity has *P* < 0.001. A threshold of eigenvalues > 1 was used as the criterion for the extraction of principal components.

## Results

In 2011 and 2021, 8,400 and 7,800 individuals aged ≥ 35 years were recruited for the study, respectively. Of these, 8,187 in 2011 and 7,572 in 2021 agreed to participate, and the response rates for the two years were 97.5% and 97.1%, respectively.

The PCA results, supported by Bartlett’s test (*P* < 0.0001) and the KMO measure (0.707), suggested that the correlations between variables were strong enough to conduct the analysis. Only the first component with eigenvalues > 1 yielded a three-component rotated solution that explained 50.8% of the total variance in the given data. The component score coefficient of education, annual household income, and access to medical services was 0.63, 0.58, and 0.65, while the low, median and high value of SEP index score was − 1.90, 0.26, and 1.42, respectively. Based on this, only one component was selected to define the SEP index, which was subsequently categorized into three levels: low, medium and high.

General characteristics of the participants are summarized in Table 1. There were no notable changes in gender composition or annual household income status between the two survey years. However, the proportion of participants aged 35–44 years in 2011 was higher compared to that in 2021 (*P* < 0.05), and the percentage of adult non-literacy declined over the study period as it was lower in 2011 than in 2021 (*P* < 0.05). The percentage of participants with good access to medical services increased from 55.5 to 67.9% (*P* < 0.05).

**Table 1:** General characteristics of the study population by survey year

Variable	Survey year	
	2011 *n* (%)	2021 *n* (%)
Sex		
Male	3960(48.4)	3739(49.4)
Female	4227(51.6)	3833(50.6)
Age		
35–44 years	1851(22.6)	1256(16.6)*
45–54 years	2119(25.9)	1905(25.2)
55–64 years	2014(24.6)	1856(24.5)
65–74 years	1403(17.1)	1670(22.1)
≥75 years	800(9.8)	885(11.7)
Ethnicity		
Han	5008(61.2)	4124(54.5)*
Minority	3179(38.8)	3448(45.5)
Education		
Illiterate	2495(30.5)	1730(22.8)*
Primary (grade 1–6) or higher	5692(69.5)	5842(77.2)
Annual household income		
Low	4196(51.3)	3844(50.8)
High	3991(48.7)	3728(49.2)
Access to medical services		
Poor	3645(44.5)	2434(32.1)*
Good	4542(55.5)	5138(67.9)
SEP		
Low	2175(26.6)	1863(24.6)*
Medium	4132(50.4)	3841(50.7)
High	1880(23.0)	1868(24.7)

*p < 0.05

Table 2 shows the age-standardized prevalence of obesity and central obesity by survey year and socioeconomic status in rural Yunnan Province. From 2011 to 2021, the general prevalence of obesity and central obesity rose from 5.9 to 12.1% and 50.2–58.0%, respectively (*P* < 0.01), and the rising rates were observed across all subcategories (*P* < 0.05 or *P* < 0.01). Both in 2011 and 2021, the prevalence of obesity and central obesity was significantly higher among females when contrasted with males (*P* < 0.01). The prevalence of both obesity and central obesity increased initially with age but subsequently decreased (*P* < 0.01), with a peak of obesity prevalence of 6.9% in 2011 and a peak of 14.4% in 2021, both in the 45–54 years old population. The prevalence of central obesity was at its highest point in 2011, reaching 53.2% among individuals aged 65–74 years old. In 2021, the prevalence increased to 61.7%, with the highest rate observed among those aged 55–64 years old. Compared to the ethnic minority population, Han ethnicity participants and individuals with poor access to medical services exhibited a higher prevalence of obesity (*P* < 0.05 or *P* < 0.01) both in 2011 and 2021. Nevertheless, the prevalence of central obesity was observed to be lower among participants of Han ethnicity compared to those of ethnic minority groups in 2011 (*P* < 0.01), and became converse in 2021 (*P* < 0.01). In both 2011 and 2021, there was a larger prevalence of central obesity observed among individuals with lower levels of education and poor access to medical services (*P* < 0.01). A positive relationship between annual household income and prevalence of obesity and central obesity was only observed in 2021 (*P* < 0.01), but prevalence of obesity and central obesity rose with SEP in both 2011 and 2021 (*P* < 0.01).

**Table 2:** Age-standardized prevalence of obesity and central obesity by survey year and socioeconomic status in rural Yunnan Province, China

Characteristic	2011				2021		
	Obesity *n*(%)		Central obesity *n*(%)		Obesity *n*(%)		Central obesity *n*(%)
Sex							
Male	191(4.7)^a^		1566(39.5)^e^		403(11.1)^af^		1907(51.3)^af^
Female	296(7.0)		2526(59.9)		504(13.2)^c^		2506(65.0)^c^
Age							
35–44 years	109(5.9)^a^		849(45.9)^a^		169(13.5)^af^		645(51.4)^ac^
45–54 years	146(6.9)		1074(50.7)		274(14.4)^f^		1103(57.9)^c^
55–64 years	122(6.1)		1039(51.6)		240(12.9)^c^		1146(61.7)^c^
65–74 years	87(6.2)		747(53.2)		167(10.0)^c^		993(59.5)^c^
≥75 years	23(2.9)		383(47.9)		57(6.4)^c^		526(59.4)^c^
Ethnicity							
Han	215(6.8)		2435(48.7)^a^		540(13.4)^af^		2522(61.0)^ac^
Minority	272(5.4)^b^		1657(52.5)		367(10.8)^c^		1891(54.5)^d^
Education							
Illiterate	132(5.3)		1309(52.4)^a^		185(10.7)^f^		1045(60.6)^bc^
Primary (grade 1–6) or higher	355(6.2)		2783(49.0)		722(12.7)^f^		3368(57.2)^c^
Annual household income							
Low	234(5.6)^af^		2073(49.6)		388(10.2)^af^		2068(53.6)^ac^
High	253(6.3)		2019(50.8)		519(14.1)^c^		2345(62.4)^c^
Access to medical services							
Poor	396(7.5)^b^		2220(51.6)^b^		643(12.6)^bf^		3038(59.4)^af^
Good	91(3.9)		1872(48.8)		264(10.5)		1375(56.2)
SEP							
Low	107(4.9)^a^		1049(47.9)^b^		194(10.4)^ac^		1225(55.7)^ad^
Medium	251(6.1)		1450(48.8)		443(11.5)^c^		1852(56.8)^c^
High	129(6.9)		1593(50.4)		270(14.5)^c^		1336(62.4)^c^
All	487(5.9)		4092(50.2)		907(12.1)^c^		4413(58.0)^c^

^a^*p* < 0.01, ^b^*p* < 0.05, ^e^*p*< 0.001, comparison of different characteristics; ^c^*p* < 0.01, ^d^*p* < 0.05, ^f^*p*< 0.001,compare with 2011

Table 3 presents the age-standardized prevalence of hypertension by survey year and socioeconomic status in rural Yunnan Province. The prevalence of hypertension showed a significant increase from 26.1 to 40.4% (*P* < 0.01) during the 10-year period under investigation, and this increasing trend was observed in all subcategories (*P* < 0.01). Both in 2011 and 2021, prevalence of hypertension increased with age (*P* < 0.01). Additionally, lower education level, annual household income, good access to medical services, and lower SEP all correlated with higher prevalence of hypertension (*P* < 0.01). A higher prevalence of hypertension was observed in women compared to men in 2011 (*P* < 0.05) but lower in women than men by 2021 (*P* < 0.01). While Han ethnicity participants exhibited higher prevalence of hypertension than ethnic minority participants in 2021 (*P* < 0.01).

**Table 3:** Age-standardized prevalence of hypertension by survey year and socioeconomic status in rural Yunnan Province, China

Characteristic	2011		2021
	hypertension *n*(%)		hypertension *n*(%)
Sex			
Male	953(25.2)^b^		1663(43.1)^af^
Female	1105(26.9)		1485(37.9)^c^
Age			
35–44 years	211(11.4)^a^		234(18.6)^ac^
45–54 years	395(18.6)		644(33.8)^f^
55–64 years	549(27.3)		857(46.2)^c^
65–74 years	563(40.1)		903(54.1)^c^
≥75 years	340(42.5)		510(57.6)^c^
Ethnicity			
Han	1296(26.7)		1841(43.7)^af^
Minority	762(25.4)		1307(36.3)^c^
Education			
Illiterate	715(28.5)^a^		797(46.6)^af^
Primary (grade 1–6) or higher	1343(25.3)		2351(38.2)^c^
Annual household income			
Low	1141(27.7)^a^		1642(42.0)^bc^
High	917(24.3)		1506(38.6)^c^
Access to medical services			
Poor	876(24.2)^a^		964(38.3)^af^
Good	1182(26.1)		2184(42.5)
SEP			
Low	653(30.0)^a^		856(45.9)^bf^
Medium	1001(24.2)		1561(40.6)^c^
High	404(21.5)		731(39.1)^c^
All	2058(26.1)		3148(40.4)^c^

^a^*p* < 0.01, ^b^*p* < 0.05, ^e^*p*< 0.001, comparison of different characteristics; ^c^*p* < 0.01, ^f^*p*< 0.001, compare with 2011

The results of multivariate logistic regression for the prevalence of hypertension by survey year in rural Yunnan Province are presented in Table 4. After adjusting for sex, age, education, ethnicity, annual household income, access to medical services, family history of hypertension, smoking, and drinking, participants who were obese and centrally obese had a higher likelihood of being hypertensive in both 2011 and 2021 (*P* < 0.01).

**Table 4:** Logistic regression for prevalence of hypertension by survey year in Yunnan Province, China

Characteristic	2011			2021	
	Adjusted odds ratio†	95% CI		Adjusted odds ratio†	95% CI
Obesity	2.98**	(2.43, 3.64)		1.99**	(1.71, 2.33)
Central obesity	1.96**	(1.75, 2.19)		1.94**	(1.75, 2.16)

**p < 0.01; †adjusted for sex, age, education, ethnicity, annual household income, access to medical services, family history of hypertension, smoking, and drinking

## Discussion

The survey results suggest an overall upward tendency across all socioeconomic groups in prevalence of obesity, central obesity, and hypertension in the rural southwest Chinese adult population. Furthermore, they highlight that both obesity and central obesity represent significant risk factors in the development of hypertension.

The prevalence of obesity in the present study was lower than that observed in the global Nutrition Report (12.3% in men and 16.2% in women) [7], studies from the United States (39.8%) [26], the latest national prevalence estimates in China (16.4%) [8], and the rural interior regions of China (16.82%) [27]. However, the prevalence of central obesity was greater than that observed in Nigeria (39%) [28], results from other rural regions of China (43.71% and 10.2%) [27, 29], and a national study of China (35.4%) [13]. A possible explanation for these relatively higher levels could be due to various factors, such as the combined influences of lifestyle habits, physical constitution, economic growth, social welfare, and cultural context. Specifically, there may be varying rates of dietary shifts taking place across various regions of China [8]. Additionally, the present study uncovered a markedly increasing trend in prevalence of obesity and central obesity. However, central obesity was more prevalent than obesity, according with earlier observations [8, 11, 30]. Due to the fact that, compared to obesity, central obesity has a stronger association with cardiometabolic risk factors, chronic disease risk, and all causes of mortality [30], governmental interventions should focus on the prevention and management of central obesity.

Based on the study conducted, it was found that over the past decade, the prevalence of obesity and central obesity was higher in women as compared to men. However, the difference in prevalence narrowed in 2021 compared to 2011 due to a trend that is picking up speed in prevalence of obesity and central obesity among men, pointing to a probable obesity crisis in the future among men in the area under investigation. This result aligns with other studies in this time period [31–33], and suggests the importance of efforts particularly tailored to prevent and control obesity in men.

The current research also revealed that those of Han ethnicity were more likely to suffer from obesity and central obesity than ethnic minorities in 2021, an ethnic difference that accords with findings in other Chinese studies [23]. Moreover, in our study, the Han ethnicity population presented a more marked upward trend in prevalence of obesity and central obesity from 2011 to 2021 than ethnic minorities. This could be attributed to a combination of genetic and environmental heterogeneity factors. The precise reasons behind the considerable ethnic differences in obesity and central obesity rates uncovered in this research need further research.

The evidence is increasingly clear that individuals with lower levels of education are more likely to have obesity and central obesity [18]. In our study low education level had a positive association with prevalence of central obesity. A possible explanation for this might be that along with urbanization, central obesity is exacerbated by a high-fat diet, sedentary lifestyle, and decreased physical activity, and in modern China, where the environment is increasingly obesogenic, individuals with higher levels of education may have greater access to resources that allow them to attain thinner body sizes [9, 34]. In addition, while the data showed an overall rise in the prevalence of obesity and central obesity across populations of all education levels throughout the period of analysis, no notable disparities in obesity and central obesity trends by education level were found. The findings underscore there is a pressing demand for health education on obesity in the study region, focusing particularly on people with less educational attainment.

Annual household income had a significant positive association with obesity and central obesity in 2021 in the current study, and the upward trend of obesity and central obesity also presented more quickly in the high-income population across the ten years study period. This discovery is consistent with findings from prior research conducted in China and other nations with low- and middle-level incomes [18, 35], and underscores an urgent need to improve health education about the hazards of obesity in populations with high earnings. Furthermore, our study indicated poor access to medical services is associated with higher odds of having obesity and central obesity both in 2011 and 2021. These inverse relationships between access to medical services and prevalence of obesity and central obesity suggest that improved access to healthcare is a crucial factor in prevention of obesity and central obesity.

The results also revealed that higher SEP is associated with an increased likelihood of developing both obesity and central obesity, and those with the most quickly increasing SEP experienced the largest increases in both prevalence of obesity and central obesity during the ten years study period. Previous studies also confirmed the prevalence of obesity and central obesity generally increased with increasing SEP in China [8, 36]. Such findings indicate that the relationship between SEP with obesity and central obesity is still in a “developing country pattern” in rural southwest China [17].

The current study revealed a significantly higher prevalence of hypertension (40.4%) compared to the national rate in China (31.5%) [13], previous studies on rural southern China (24.0%) [37], and other low- and middle-income countries (32.3%) [38]. Furthermore, a notably rising trend in prevalence of hypertension was uncovered in 2021 (40.4%), as compared to 2011 (26.1%), across all socioeconomic gradients. These findings are consistent with other rural regions in China [39, 40]. This rising trend is likely related to increases in urbanization, dietary changes, and obesity, as well as an aging population [41]. The findings highlight that hypertension remains a noteworthy, and expanding public health issue in rural Southwest China that demands intervention in this region. Further, our study revealed a change in the distribution of hypertension prevalence according to gender over the study period, with male hypertension increasing at a faster pace than females from 2011 to 2021. This is possibly due to the fact that smoking, drinking, and high-salt and high-fat diets are increasingly more common in males than females [14]. Additionally, males had a faster increase in obesity and central obesity than females over the study period. Future hypertension and obesity prevention measures should thus focus in particular on males.

The results indicated that obese and centrally obese participants were more likely to be hypertensive both in 2011 and 2021. Weight gain has been consistently linked to an increase in blood pressure according to numerous studies, and obesity has been recognized as an independent, modifiable risk factor for hypertension [42]. Our findings highlight the harmful outcomes that may arise in the future due to the ongoing epidemics of obesity and central obesity. Additionally, we observed a decrease in the strength of the link between obesity and central obesity with hypertension over time. This finding aligns with previous researches in Germany, Seychelles, and England [43, 44], but contrasts with findings in the US and Japan, where the link between obesity and hypertension has been more pronounced today than it was in past decades [45]. Obesity and obesity-related hypertension involve intricate and sometimes interconnected mechanisms [46]. This could be due to advancements in hypertension diagnosis and treatment [43, 44]. Another possible explanation is the increasing prevalence of other determinants for hypertension [44]. Further research is needed to identify the reasons behind the changing correlation between obesity and central obesity with hypertension over time.

The present study not only provides an opportunity to assess long-term changes in obesity, central obesity, and hypertension, but changes in the association between obesity and central obesity with hypertension over time. The study findings, however, are constrained in three respects. First, dietary intake and physical activity data were not collected in this study. Therefore, we were unable to determine associations between these factors and the changes in the prevalence of obesity, central obesity, and hypertension observed. Second, causal relationships could not be established as the data for this study was obtained from cross-sectional survey. Third, we only used BMI to measure adiposity, due to BMI is merely a proxy for fatness, we could not directly address aspects of body composition such as fat distribution or visceral fat. Moreover, the definition of obesity with BMI ≥ 28 kg/m2 may have an excess BMI due to high muscle mass rather than true excess adiposity, so the true prevalence of obesity is likely overestimated.

## Conclusions

In conclusion, the findings indicate that over the ten years studied, obesity, central obesity, and hypertension not only remained at a high level but have increased substantially in rural China, with SES an important influence on temporal trends for the observed rates. Future obesity interventions in rural China should give increased attention to high-income, less educated, poor access to medical services, and high SEP individuals, as well as those of Han ethnicity. The implementation of obesity interventions would also help reduce morbidity associated with hypertension.

## Data Availability

The datasets used and/or analyzed in this study are available from the corresponding author on reasonable request.

## Abbreviations

AHA: American Heart Association
BMI: Body Mass Index
BP: Blood Pressure
CI: Confidence Interval
CHNS: China Health and Nutrition Survey
GDP: Gross Domestic Product
KMO: Kaiser-Meyer-Olkin
PCA: Principal Components Analysis
PPS: Probability Proportional to Size
NCDs: Non-communicable Diseases
SEP: Socioeconomic Position
WC: Waist Circumference
WHO: World Health Organization
